# Multi-Sensor Assessment of Pigeon Flight Behavior: Role of Biomechanical and Landscape Characteristics

**DOI:** 10.3390/s26030916

**Published:** 2026-01-31

**Authors:** Flavia Forconi, Ilenia De Meis, Giacomo Dell’Omo, Valentina Camomilla, Giuseppe Vannozzi, Maurizio Schmid, Silvia Conforto, Daniele Bibbo

**Affiliations:** 1 Department of Industrial, Electronic and Mechanical Engineering, Roma Tre University, 00146 Rome, Italy; ilenia.demeis@uniroma3.it (I.D.M.); maurizio.schmid@uniroma3.it (M.S.); silvia.conforto@uniroma3.it (S.C.); daniele.bibbo@uniroma3.it (D.B.); 2 Ornis italica, 00199 Rome, Italy; giacomo.dellomo@gmail.com; 3 Department of Movement, Human and Health Sciences, University of Rome “Foro Italico”, 00135 Rome, Italy; valentina.camomilla@uniroma4.it (V.C.); giuseppe.vannozzi@uniroma4.it (G.V.)

**Keywords:** homing pigeons, wearable devices, accelerometer, wingbeat frequency, biologging, time-frequency analysis, altitude, land cover classification

## Abstract

Understanding how birds adjust their flight in response to biomechanical characteristics and environmental conditions can be useful for interpreting homing behavior. This study investigates homing pigeons’ (*Columba livia*) flight behavior using multi-sensor biologgers, integrating GPS, tri-axial accelerometer, pressure, and temperature sensors. Flight biomechanics were assessed by extracting: wingbeat frequency from the Short-Time Fourier Transform of the total acceleration signal and peak-to-peak acceleration from the dorso-ventral component. Landscape characteristics were provided by classifying land cover along the route using a geographic atlas and by computing flight altitude above ground level through the combination of pressure-derived altitude and a digital elevation model. The results reveal a progressive decrease in wingbeat frequency along the homing route, showing a linear relationship with traveled distance. To assess whether this pattern can be interpreted in terms of flight regulation, flight altitude was modeled as a function of biomechanical and environmental variables using a linear mixed-effect approach. The analysis indicates that flight altitude is significantly affected by wingbeat frequency as well as by temperature, ground speed, and land cover, with wingbeat frequency and temperature showing the strongest negative association.

## 1. Introduction

Wearable sensors have become an established tool for the continuous, non-invasive monitoring of physiological and behavioral parameters, as well as environmental conditions in a variety of animal species [1,2]. Advances in miniaturized electronics have enabled the development of compact devices capable of integrating multiple sensors, allowing for long-term data collection with minimal disturbance to the subject [3,4]. In the context of biologging, i.e., the use of animal-borne electronic devices (biologgers) to study wildlife ecology, behavior, physiology, and environmental interactions [5,6], such devices can now record several variables, such as acceleration, GPS location, air pressure, and air temperature, at high frequency over extended periods of time [7,8]. For instance, the investigation of pigeons’ flight behavior has been facilitated by means of these biologgers, primarily including GPS data loggers [9] and high-frequency tri-axial accelerometers [10,11,12], which are directly attached to the birds. These biologgers enable the detailed reconstruction of flight trajectories and allow for precise measurement of flight dynamics in free-ranging pigeons in relation to environmental and landscape characteristics [5,13].

For the comprehension of the biomechanical flight characteristics, such as flight control and energetic expenditure, wingbeat dynamics play a central role since the primary determinants of aerodynamic power for a flapping wing are wingbeat frequency and amplitude [14]. Wingbeat characteristics have been investigated in relation to several behavioral and environmental conditions [15,16]. For instance, a couple of seminal papers [15,17] studied the biomechanical cost of flying and observed that flying in a flock increases energy expenditure with respect to flying solo. Tobalske et al. [16] investigated in depth the flight kinematics of black-billed magpies and pigeons flying at different speeds in a wind-tunnel. In pigeons, the authors observed that wingbeat frequency remains largely invariant across increasing flight speeds, with speed modulation achieved through adjustments in overall flight mechanics (e.g., body posture, aerodynamic gait) rather than in flapping rate. Furthermore, the variations in the wingbeat characteristics of homing pigeons were analyzed as they learned the homing task, demonstrating that birds modulate these characteristics as a function of their navigational knowledge [18]. However, these studies have primarily treated wingbeat characteristics as temporally averaged variables rather than examining how they vary along the spatial dimension of a flight path. To the best of our knowledge, a time–frequency-based quantitative assessment of the spatial distribution of wingbeat frequency along a long-distance continuous homing flight has not yet been reported. This analysis can potentially allow us to relate the biomechanical variations in homing pigeons’ flight to landscape and environmental changes. For instance, altitude regulation represents a key challenge during sustained flights across heterogeneous landscapes. Terrain features such as urban areas and non-urban ones (e.g., forests, agricultural fields, and water bodies) can create distinct airflow and thermal conditions, potentially affecting how birds adjust their movement during flight (e.g., change in altitude) [19]. Moreover, environmental variables such as air temperature and pressure may influence flight altitude and, therefore, wingbeat patterns. To the best of our knowledge, studies on the interaction between flight biomechanics and landscape characteristics have predominantly focused on migratory birds, as their sustained long-distance flights provide an ideal context to investigate biomechanical and environmental interactions [20,21].

Despite the lack of migratory behavior in their natural history, homing pigeons, taking advantage of their strong homing instinct and capacity to return to their loft from unfamiliar locations [22], constitute an effective model for investigating flight biomechanics, also in relation to landscape and environmental conditions. Garde et al. [23] analyzed fine-scale changes in flight speed and altitude in homing pigeons released approximately 6 km from their loft, showing that birds employ high power output to return rapidly, resulting in energetically inefficient flight characterized by variations in altitude, speed, and trajectory. Moreover, the authors showed that homing pigeons exhibit fine-scale modulation of flight altitude and speed in relation to underlying topography, with systematic altitude changes associated with hills and valleys along the route, indicating a close coupling between vertical flight behavior and landscape structure. More recent studies have demonstrated that environmental factors, such as atmospheric turbulence, can influence wingbeat patterns in free-flying pigeons, inducing adjustments in wingbeat dynamics and increasing variability in flapping behavior in response to unsteady airflow [24,25]. However, studies conducted on homing pigeons’ flight dynamics have focused on relatively short homing routes [17,18,23], with trajectories spanning less than 10 km, and the wingbeat frequency has not been considered as a biomechanical descriptor of the homing flight in combination with the landscape characteristics (e.g., altitude). Therefore, the relationship between wingbeat dynamics, with particular attention to wingbeat frequency, and landscape characteristics, including flight altitude and its maintenance, remains unexplored in homing pigeons during long-distance flights.

To address this gap, the present study aims at understanding how homing pigeons adjust their flight in response to biomechanical characteristics and environmental conditions, by adopting a two-step approach. First, a quantitative and descriptive analysis of wingbeat frequency along the homing route is provided to investigate its distance-dependent pattern. Second, this pattern is evaluated to determine whether it can be mechanistically interpreted in terms of flight altitude regulation, by modeling altitude as a function of biomechanical and landscape characteristics. To do this, a multi-sensor assessment of homing pigeons’ flight behavior was conducted, evaluating biomechanical and landscape characteristics. Pigeons, released at 18 km from their loft, were equipped with GPS, tri-axial accelerometer, pressure, and temperature sensors to capture detailed flight dynamics during the homing flight. Wingbeat frequency and peak-to-peak acceleration were used as indicators of flight modulation, while landscape features along the flight path were classified using a geographic atlas. This approach enables a detailed, spatially resolved analysis of wingbeat dynamics and altitude regulation, providing new insights into the interaction between flight biomechanics, environmental conditions, and landscape heterogeneity during navigation, thus providing insights into how homing pigeons adjust their flight in response to both internal and external factors.

## 2. Materials and Methods

### 2.1. Subjects and Experimental Campaign

Seven adult homing pigeons of both sexes (*Columba livia*) were considered in this study (weight: 440 ± 59 g). The subjects were part of the same loft located near Colleverde, Rome, Italy (41.58° N, 12.37° E). Access to unlimited water and food was always ensured in the loft and all subjects were permitted to take daily free flights around the loft from sunrise to sunset. In addition, all birds had prior homing experiences to familiarize themselves with the general surrounding environment. Two weeks prior to the start of the experimental campaign, a basic homing training phase was conducted. The pigeons were equipped with a plastic dummy comparable in mass and size to the actual data logger (approximately 12 g, <3 % of the body mass of the pigeon). It was attached to the pigeon’s back with Velcro strips and a biocompatible and removable glue to guarantee firm but non-invasive contact [9]. This phase was essential to ensure that the birds were accustomed to carrying an additional load during daily free flights and to minimize any behavioral artifacts due to the equipment during experimental trials.

**Figure 1:**
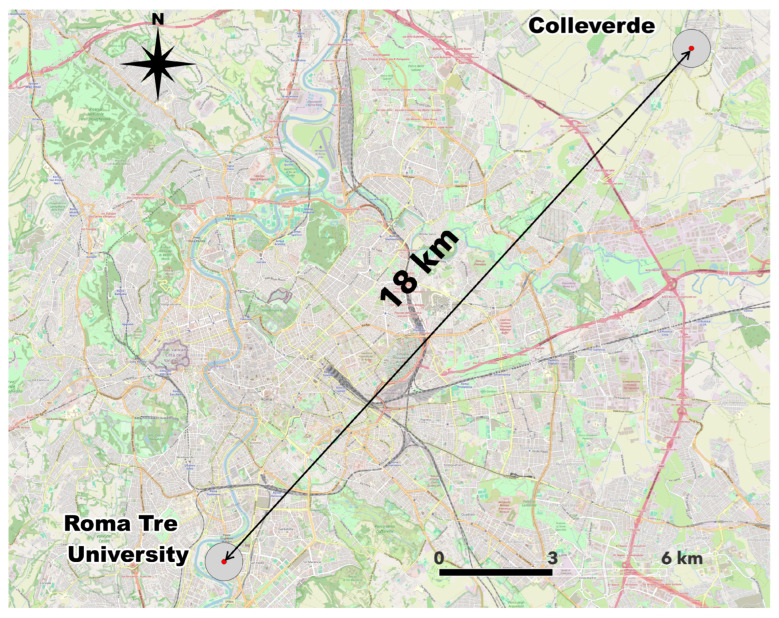
Distance of homing pigeons’ flight path from Roma Tre University to the loft located in Colleverde. Map designed using QGIS 3.36.1.

The experimental campaign consisted of homing pigeons’ releases from the Roma Tre University Campus (41.51° N, 12.28° E), which is an urban site located approximately 18 km in a straight line from the loft (Figure 1). The releases were conducted between April and May 2024 on days with favorable meteorological conditions (clear skies, no precipitation, and negligible wind) to limit environmental confounding factors. Specifically, the releases were conducted in the morning between 10:00 a.m. and 12:00 p.m. local time, with pigeons being released simultaneously. All pigeons successfully completed their home flight and returned to the loft on the same day. From the database of the collected data, only the records corresponding to continuous flights (i.e., no stops between the initial and final sites) were selected, thus obtaining a sample of 13 tracks.

### 2.2. Data Logging

**Figure 2:**
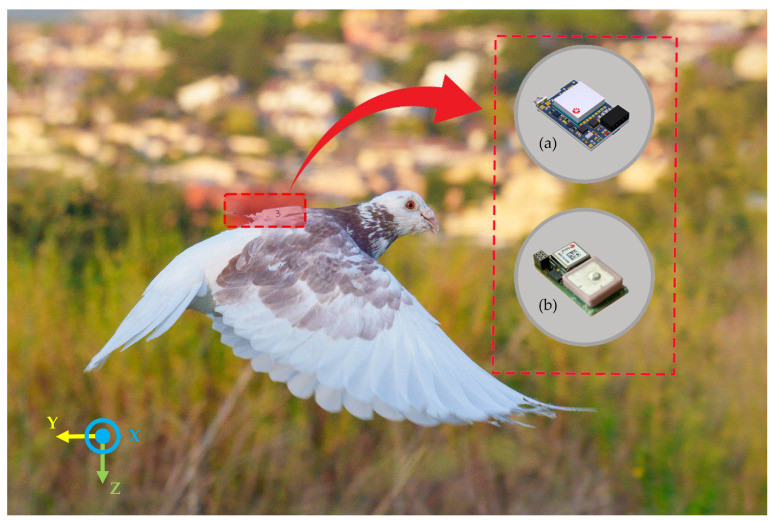
A homing pigeon (*Columba livia*) equipped with the Axy-Trek data logger composed of a tri-axial accelerometer (**a**) and a GPS logger and environmental sensors (**b**).

The pigeons were tracked using a commercial data logger (Axy-Trek, Technosmart Europe, Rome, Italy), which included a tri-axial accelerometer, a GPS module and sensors for air temperature (°C) and pressure (mbar) (Figure 2). The sampling frequency of the tri-axial accelerometer was set at 50 Hz with a chosen range of ±4 g, while for the other sensors, it was set at 1 Hz.

The GPS logger provided the ground speed ([km/h]), that is, the speed of the bird projected on the ground, and the GPS data were also used to obtain the distance traveled from the release site. Concurrently, the altitude above mean sea level (ASL [m]) was measured by means of a dedicated pressure sensor [26]. The three axial components of the accelerometer were medio-lateral (*x*-axis), cranio-caudal (*y*-axis), and dorso-ventral (*z*-axis), as reported in Figure 2. On the day of the experiment, each plastic dummy was replaced by a data logger approximately 15 min before the release. Devices were retrieved when pigeons returned to the loft and data were downloaded using X-Manager 1.23 software (@Technosmart Europe, Italy) for post-processing.

### 2.3. Data Processing

**Figure 3:**
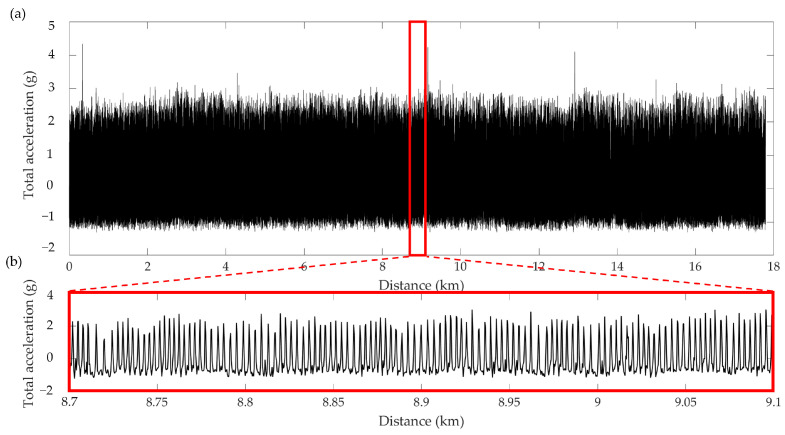
Total acceleration during a flight as a function of the traveled distance (**a**) and a detail of approximately 400 m (**b**).

The initial ascending phase immediately following the release and the final descending phase to the loft were excluded from the analysis. These phases were determined by using the coordinates obtained through the GPS and all the points within a circular area of a 500-m radius around the initial and final sites were excluded. Subsequent analyses were performed on flight tracks segmented according to this procedure. For synchronization purposes, data were resampled to the sampling frequency of the accelerometer (50 Hz). The distance traveled from the release site was achieved from GPS data using the great-circle distance formula, referenced to the World Geodetic System 1984 (WGS84) reference ellipsoid [27]. To ensure independence from axis-specific gravitational components, the total acceleration (i.e., Vectorial Dynamic Body Acceleration, VeDBA [28]) was computed as the modulus of the three axial components (*x*-axis, *y*-axis, *z*-axis) and its mean value was removed. An example of the trend of total acceleration as a function of the traveled distance is shown in Figure 3 with a detail of approximately 400 m.

**Figure 4:**
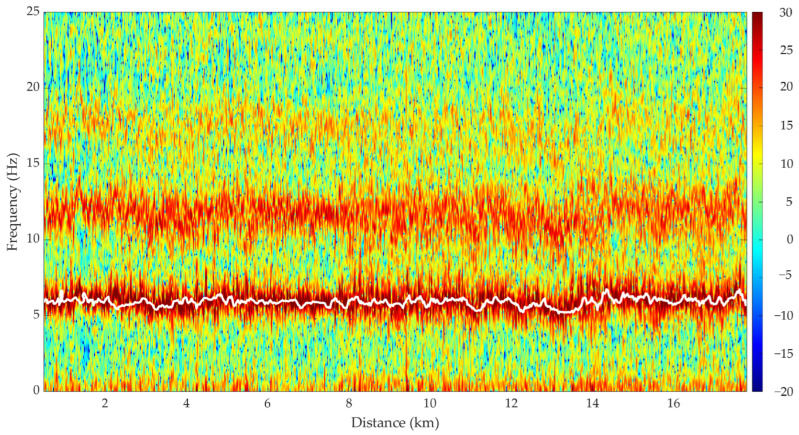
Example of the extraction of wingbeat frequency time trend (white line) from the Short-Time Fourier Transform (STFT) of the total acceleration.

The total acceleration was used to detect the wingbeat frequency during continuous and steady homing flights. Since the total acceleration of the pigeons’ flight is non-stationary, the analysis was carried out in the time–frequency domain [14,29], by using the Short-Time Fourier Transform (STFT). STFT was implemented using a Kaiser window 128-sample long sliding on the data series using a 64-sample overlap. Zero-padding to 1024 points was chosen. The wingbeat frequency was obtained by identifying the frequency values corresponding to the maximum power of the STFT of the total acceleration signal [30], in the sub-band between 3 and 9 Hz, since the wingbeat frequency is known to be approximately 5 Hz [14]. Among the visible components, the analysis was focused on the latter, postponing to future studies the investigation of the others (potentially harmonic components). Figure 4 shows an example of the wingbeat frequency extraction as a function of the distance from the release point.

In addition to wingbeat frequency, peak-to-peak acceleration was computed as an additional measurement of flight dynamics. The dorso-ventral (*z*-axis) acceleration signal was used for the detection of peak-to-peak variations, representing the amplitude of wingbeat oscillations. This was achieved by identifying the upper reversal points in acceleration, corresponding to the highest points in each wingbeat cycle [31].

To visualize the spatial variation in wingbeat frequency along the homing flight path, a distance-based radial binning approach was applied to the segmented tracks. Specifically, based on latitude and longitude values, the flight trajectories were partitioned into concentric circular bins centered on the initial position of the track. For each specific bin, the mean and standard deviation (SD) value of wingbeat frequency were computed using all samples falling within that bin, pooling data across all individuals and flights. The resulting wingbeat frequency values were subsequently mapped onto the flight trajectories and represented using a continuous color scale, allowing for visualization of distance-dependent spatial patterns in wingbeat frequency and highlighting its spatial distribution across the pigeons’ flight paths.

Terrain elevation data were obtained from the Digital Elevation Model (DEM) (10 m resolution; source: Copernicus Data Space Ecosystem), which provides surface elevation above mean sea level at high spatial resolution. For each GPS location, the corresponding DEM value was extracted to represent the local ground elevation. Flight altitude above ground level (AGL [m]) was then computed as the difference between the ASL and the DEM-derived ground elevation, resulting in an estimate of true flight height relative to the underlying terrain. This AGL value was used as flight altitude in the following analyses.

The Urban Atlas Land Cover/Land Use 2018 dataset was used for land classification [32]. For the purposes of this study, the original land cover classification was organized into two categories. Specifically, the 17 urban land classes were aggregated into a single urban class, while the 10 rural classes were combined into a single non-urban class. To account for landscape heterogeneity at the spatial scale relevant to flight behavior, a spatial averaging procedure was applied: for each 500-m flight segment, the mean value of the reclassified land cover was calculated. This resulted in a continuous urbanization index ranging from 0 (entirely non-urban landscape) to 1 (entirely urban landscape), with intermediate values representing mixed environments.

To evaluate which biomechanical and landscape characteristics influence altitude during flight, a linear mixed-effect model (LME) was used, with ground speed, wingbeat frequency, peak-to-peak acceleration, temperature, and land cover (urbanization index) as predictors. Since flight altitude was measured by means of the dedicated pressure sensor, pressure was not included as a predictor in the model. All the variables used in the LME model were preprocessed as the wingbeat frequency, previously explained. Specifically, a spatial binning at intervals of 500 m was applied: for each 500-m segment along the flight path, the mean value of each variable was computed using all samples falling within that segment and across all tracks. This preprocessing step was applied to reduce spatial and temporal autocorrelation among successive observations along the flight path, thereby better satisfying the independence assumptions underlying linear mixed-effect modeling. This scale represented the best compromise between spatial resolution of the data and smoothness empirically determined after a series of preliminary tests. Flight identity was included as a random intercept to account for the non-independence of repeated 500-m spatial segments belonging to the same homing flight. This structure models within-flight correlation and captures between-flight variability across different flight tracks. Potential multicollinearity among fixed-effect predictors was assessed by computing Pearson’s correlation coefficients and variance inflation factors (VIFs). These diagnostics were used to verify that biomechanically related predictors could be jointly included without compromising model stability.

Data were processed in MATLAB r2023b and Python 3.8 on a 12th Gen Intel Core i7 CPU, with 64 GB of RAM.

### 2.4. Statistical Analysis

The Shapiro–Wilk normality test was performed for wingbeat frequency. Kruskal–Wallis tests were performed to assess the presence of significant differences in wingbeat frequency over distance from the release site. This was followed by a post hoc analysis to identify differences between the wingbeat frequency values at different distances from the release site, using Dunn’s test for multiple-comparison correction. Statistical analysis was performed with GraphPad Prism 8.0 with α level set to 0.05. The statistical model, i.e., the LME, was applied using the statsmodels 0.14.6 Python package [33].

## 3. Results

**Figure 5:**
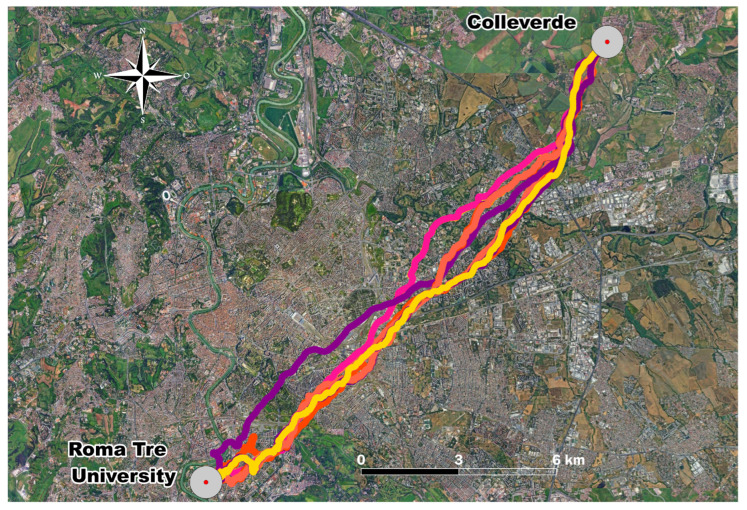
Homing pigeons’ flight paths of the 13 tracks from Roma Tre University to the loft located in Colleverde. Each color line represents a single track. Map designed using QGIS 3.36.1.

The 13 flight tracks analyzed were recorded from pigeons flying in flock during the different release days. Figure 5 shows the distinct trajectories overlaid on the map, together with the 500 m exclusion zones marked around the release and arrival sites. Following segmentation to exclude the initial and final 500 m of the flight, the remaining tracks were divided into consecutive 500 m bins along the flight path, resulting in a total of 35 segments.

**Figure 6:**
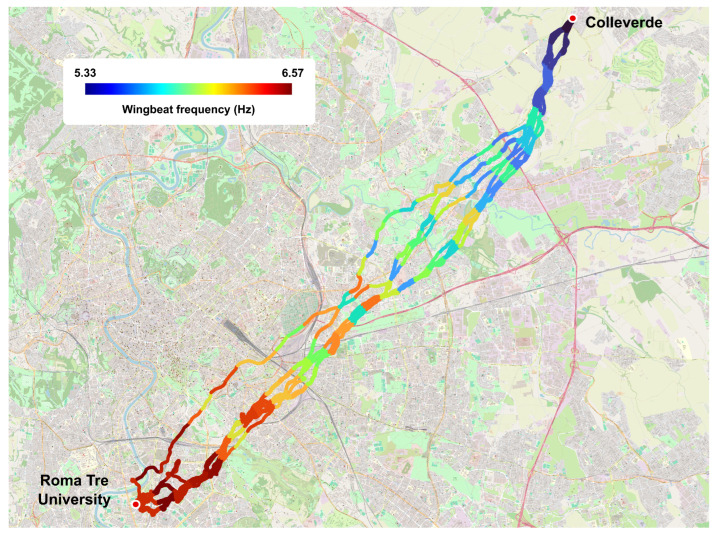
Distance-based radial binning of the mean wingbeat frequency of the 13 tracks along the homing pigeons’ flight path from Roma Tre University to the loft located in Colleverde. Map designed using QGIS 3.36.1.

Figure 6 shows the distance-based radial binning of the mean wingbeat frequency along the homing pigeons’ flight path from Roma Tre University to the loft located in Colleverde.

It is worth noting that wingbeat frequency decreases along the homing route. For instance, near the release site, the wingbeat frequency is approximately 6.57 Hz, while near the loft, it is about 5.33 Hz, representing a decrease of approximately 19%.

**Figure 7:**
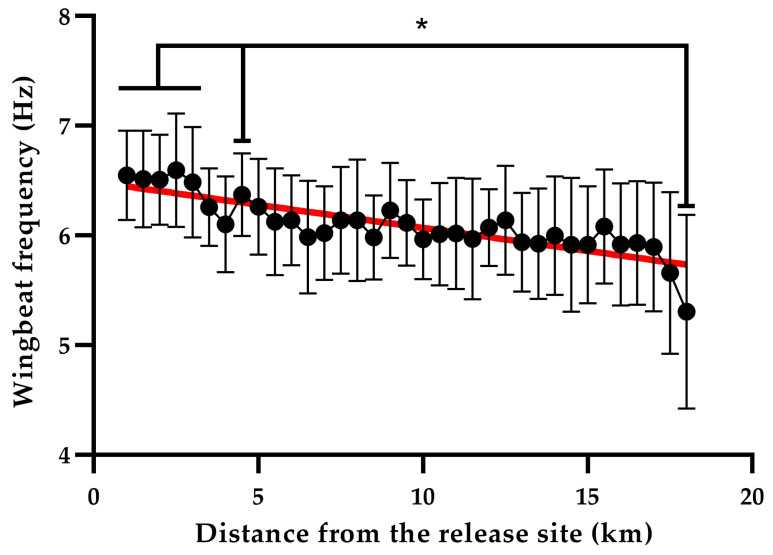
Trend of the wingbeat frequency as a function of the distance from the release site. Values mean ± SD. *: *p*-value < 0.05. The red line corresponds to the linear regression fitting.

Figure 7 shows the trend of the mean wingbeat frequency for each bin as a function of the distance from the release site, along with the corresponding relative linear fitting.

The Kruskal–Wallis test reveals a significantly higher wingbeat frequency within 3 km from the release site, in comparison to the last value, in proximity of the loft. A further significant difference is identified between the wingbeat frequency value at 4.5 km from the release site and the last one. Furthermore, the linear regression analysis yields a determination coefficient $R^{2}$ = 0.71, demonstrating a linear decrease of the wingbeat frequency as a function of the distance covered by the homing pigeons during their return flight.

**Figure 8:**
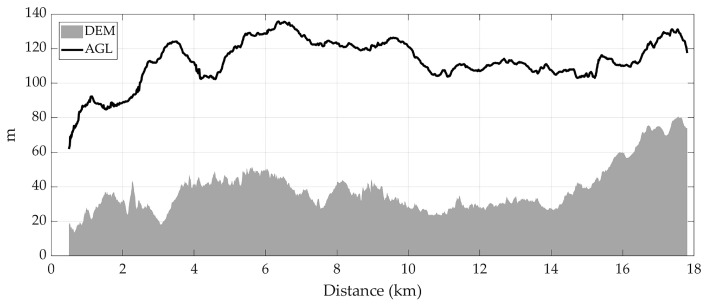
Mean flight altitude above ground level (AGL) changes along homing route in relation to the underlying Digital Elevation Model (DEM).

As regards landscape characteristics, Figure 8 shows the mean flight altitude changes across all tracks along homing route in relation to the underlying DEM. The DEM trend reveals significant terrain heterogeneity, with elevation gradually increasing towards the end of the route. In contrast, despite these changes in topography, the AGL varied between 60 and 88 m during the homing flight path. While there are some fluctuations in flight altitude, particularly in the first half of the route, the overall pattern indicates that the birds regulate their flight height to maintain a consistent distance above the ground. Towards the end of the route, as the terrain becomes steeper, the birds increase their altitude accordingly to preserve a similar AGL.

Table 1 shows the results of the LME model, showing the effect on flight altitude with outcome and ground speed, wingbeat frequency, land cover, peak-to-peak acceleration and temperature as predictors. Variance inflation factors were low for all predictors (VIF < 2), indicating no problematic multicollinearity and supporting their simultaneous inclusion in the models. The model was executed with standardized variables to compare the magnitude of their effects, while raw estimates allow for quantitative interpretation of their effects. Ground speed, wingbeat frequency, land cover and temperature overall exhibited a significant effect on flight altitude. The linear mixed-effect model reveals a significant intercept. Temperature shows the largest standardized effect, and it is negatively related to flight altitude ($\beta_{std}$ = −1.205, ${SE}_{std}$ = 0.180, z = −6.710, *p* < 0.05). Wingbeat frequency is strongly and negatively related to altitude, indicating that changes in altitude are associated with variations in wingbeat frequency. An increase of 1 Hz in wingbeat frequency was associated with a significant decrease of 23.09 m in altitude ($\beta_{unstd}$ = −23.091, ${SE}_{unstd}$ = 4.649, z = −4.967, *p* < 0.05). Land cover effects reveal a significant change in flight altitude depending on land cover type (urban or non-urban). Land cover was found to be positively related to flight altitude, with altitude increasing in urban context and decreasing in non-urban one. Ground speed has a positive and statistically significant effect on the flight altitude ($\beta_{std}$ = 0.092, ${SE}_{std}$ = 0.038, z = 3.380, *p* = 0.001), indicating that higher ground speed is associated with higher values of the dependent variable. An increase of 1 km/h of ground speed is associated with an increase of around 0.51 m of altitude, statistically significant ($\beta_{unstd}$ = 0.508, ${SE}_{unstd}$ = 0.150, z = 3.380, *p* < 0.05). In contrast, peak-to-peak acceleration does not have statistically significant effects on the altitude.

## 4. Discussion

This study provides a multi-sensor assessment of pigeon flight behavior, focusing on flight dynamics and landscape characteristics.

Wingbeat frequency is computed through a time–frequency analysis of the total acceleration. While previous studies have, in general extracted wingbeat frequency from accelerometer signals in the time domain [14,18], our approach takes into account the non-stationary nature of the signal. This allows for continuous monitoring of wingbeat frequency over time, and therefore over the distance traveled by the pigeons, providing a comprehensive perspective on the biomechanical behavior of birds during the homing process. It is worth noting that all flight tracks were recorded while pigeons were flying in a flock. Social interactions and collective flight dynamics may influence both wingbeat frequency and altitude regulation, potentially constraining individual biomechanical responses [15,17,34]. Future studies should compare solo and flock flights to disentangle individual from group-level effects.

This study provides strong evidence that wingbeat frequency decreases during the flight from the release site towards the loft. Additionally, regression analysis shows a linear decrease in wingbeat frequency with increasing distance from the release site during the return flight. These results indicate that homing pigeons actively modulate their wingbeat rate in relation to the remaining distance to their destination. Such modulation can be interpreted as a biomechanical strategy aimed at optimizing energy expenditure [14,18], potentially in interaction with landscape characteristics [34]. The spatial distribution of wingbeat frequencies further supports this interpretation.

However, distance per se does not provide a mechanistic explanation for this pattern, as it does not directly constrain wingbeat kinematics. Distance from the release site instead covaries with several flight-related parameters that change systematically along the homing route, including flight altitude and the spatial context through which birds travel. Distance-related fatigue is unlikely to explain the observed decrease in wingbeat frequency, as fatigue in homing pigeons has been reported only after much longer flights (approximately 3 h or 60 km) [35,36], well beyond the distance covered in the present study (approximately 18 km). Therefore, it is reasonable to exclude fatigue as a possible cause of the observed distance-frequency pattern.

As pigeons progress toward the goal, they adjust their flight altitude in response to environmental conditions and landscape features, all of which can influence aerodynamic and biomechanical demands. Flight altitude was therefore treated as an integrative variable to evaluate whether changes in wingbeat frequency are functionally associated with altitude regulation driven by biomechanical and environmental factors. The LME model was used as a tool to evaluate consistency between observed wingbeat modulation and known mechanisms of altitude control in flapping flight. The model indicates a strong negative relationship between wingbeat frequency and flight altitude, with lower wingbeat frequencies associated with higher flight altitudes. This result suggests that the gradual decrease in wingbeat frequency during homing may be partially associated with changes in flight altitude, rather than being a function of distance alone. Moreover, ground speed was positively associated with altitude, and this relationship is consistent with aerodynamic principles, as higher speeds may facilitate climbing or maintaining flight at greater altitudes with reduced energetic cost. Also, higher altitudes were associated with lower air temperature, likely reflecting multiple interacting factors: higher temperatures reduce air density, lowering aerodynamic lift and increasing energetic costs, which may favor flying at higher flight altitudes [21,37]. Additionally, thermal updrafts are often closer to the ground during warm conditions, and physiological constraints such as metabolic and thermoregulatory limits may further restrict low-altitude flight. Together, these factors suggest that temperature effects on flight altitude are context-dependent and mediated by both atmospheric and physiological mechanisms. Land cover also exerted a significant effect on altitude, highlighting the role of environmental features in shaping flight behavior. Pigeons increased flight altitude when flying above urban areas, possibly due to the presence of buildings and other obstacles, disturbance or noise. This supports the idea that pigeons integrate biomechanical and landscape-related cues to optimize their flight, adjusting altitude in response to underlying terrain characteristics, potentially to improve navigational efficiency or energetic performance. In contrast, peak-to-peak acceleration did not significantly change with altitude. However, when flying at lower altitude, pigeons exhibited a significant increase in wingbeat frequency, accompanied by a non-significant decrease in peak-to-peak acceleration. This pattern suggests that altitude regulation primarily relies on modulation of wingbeat frequency rather than acceleration amplitude. This interpretation is consistent with biomechanical and energetic models indicating that wingbeat frequency contributes more strongly to lift and power modulation than wingbeat amplitude, given that inertial power requirements (i.e., cost of steady flight) scale with the square of wingbeat amplitude and the cube of wingbeat frequency [18].

Taken together, these results highlight the importance of a multi-sensor approach for understanding the complex interactions between biomechanical variables and environmental features in avian flight. The coordinated modulation of wingbeat frequency, altitude, and ground speed suggests that pigeons dynamically adjust their flight mechanics in response to both internal energetic constraints and external landscape characteristics during homing. As a potential extension offered by the time–frequency analysis, further insights about the behavior of the pigeons could be obtained from a deeper analysis of the harmonic components, which is out of the scope of the present study.

An important direction for future research concerns the potential influence of air quality on flight altitude and biomechanical flight parameters. In particular, atmospheric pollutants such as CO_2_ and other airborne compounds may affect flight performance either directly, through physiological constraints, or indirectly, by modifying air density or local aerodynamic conditions. Incorporating onboard air-quality sensors into the multi-sensor framework would allow for continuous measurements of pollutant exposure during flight and enable the assessment of their effects on altitude, wingbeat frequency, and ground speed. The inclusion of air pollution metrics as additional predictors within the linear mixed-effect modeling framework could provide new insights into how environmental conditions interact with biomechanical and navigational strategies during homing flight, also using large language models approach [38]. Such an approach would further enhance the integrative nature of the present study and contribute to a more comprehensive understanding of how anthropogenic factors influence avian flight behavior in heterogeneous landscapes.

## 5. Conclusions

The objective of this study is to provide a comprehensive multi-sensor assessment of pigeon flight behavior through the description of biomechanical and landscape characteristics, offering novel insights into how pigeons adjust their flight in response to both internal and external factors. The findings of this study reveal that pigeons modulate wingbeat frequency and altitude during the homing route, suggesting an energy management optimization. Changes in flight altitude are associated with variations in wingbeat frequencies. The interaction between altitude and urban landscapes indicates that pigeons adapt their flight behavior in response to environmental heterogeneity and constraints.

Future research should integrate air-quality sensors into multi-sensor models to evaluate how atmospheric pollutants influence flight biomechanics, thereby improving the understanding of the interactions between anthropogenic environmental factors, avian navigation, and energy management.

## Figures and Tables

**Table 1:** Statistical results of the LME model showing the effects on flight altitude of ground speed, wingbeat frequency, land cover, temperature, and peak-to-peak acceleration. The model was applied with standardized (std) and unstandardized (unstd) variables. S.E. indicates Standard Error.

Variable	Coefficient (std)	S.E. (std)	Coefficient (unstd)	S.E. (unstd)	z	*p*-Value
Intercept	0.000	0.457	136.639	33.176	4.119	0.000
Ground speed	0.092 ^∗^	0.038	0.508 ^∗^	0.150	3.380	0.001
Wingbeat frequency	−0.246 ^∗^	0.057	−23.091 ^∗^	4.649	−4.967	0.000
Land cover	0.128 ^∗^	0.032	21.527 ^∗^	5.432	3.963	0.000
Temperature	−1.205 ^∗^	0.180	−14.808 ^∗^	2.207	−6.710	0.000
Peak-to-peak acceleration	0.013	0.080	7.980	8.793	0.908	0.364

* *p*-value < 0.05.

## Data Availability

The original contributions presented in this study are included in the article/Supplementary Materials. Further inquiries can be directed to the corresponding author.

## Supplementary Materials

The following supporting information can be downloaded at: https://www.mdpi.com/article/10.3390/s26030916/s1.

## Abbreviations

The following abbreviations are used in this manuscript:

GPS	Global Positioning System
STFT	Short-Time Fourier Transform
ASL	Altitude above mean sea level
AGL	Altitude above ground level
DEM	Digital Elevation Model
LME	Linear mixed-effect model
SD	Standard deviation
VIF	Variance inflation factor
